# Institutional Experience With Surgical and Injectable Procedures at a Department Resident Aesthetic Plastic Surgery Clinic

**DOI:** 10.1093/asjof/ojad027.005

**Published:** 2023-04-14

**Authors:** Zachary Borab, Carter Boyd, Hani Nassir, Michael Cassidy, Neil Vranis, Alexis Gursky, Rebecca Gober, Barry Zide, Daniel Ceradini

**Affiliations:** New York University, New York, NY; New York University, New York, NY; New York University, New York, NY; New York University, New York, NY; New York University, New York, NY; New York University, New York, NY; New York University, New York, NY; New York University, New York, NY; New York University, New York, NY

## Abstract

**Goals/Purpose:**

Over the past several decades, the number of aesthetic procedures in plastic surgery has risen drastically in the United States. To meet this public demand, it is important to ensure plastic surgery residents are well-equipped to incorporate aesthetics into their future practice. Given the elective nature of aesthetic surgery, many patients opt to personally select their surgeon from qualified private practitioners. Patients’ tendency to avoid academic institutions, coupled with relatively high postoperative expectations, has historically led to limited hands-on experience for residents. Recent literature has documented graduating plastic surgery residents have felt deficient in performing aesthetic procedures, particularly those of the face. In a recent survey, 30% of department chairs/division chiefs acknowledged difficulties with and inadequacies of aesthetic surgery training at their institutions and recommending the need for more effective training models. The objective was to review the volume and outcomes of our resident aesthetic clinic.

**Methods/Technique:**

The Resident Aesthetic Surgery Clinic at the Hansjörg Wyss Department of Plastic Surgery at NYU Langone Health sees patients 5 days per week and provides a total of 6 months of training experience for residents. Patients presenting to the clinic are first evaluated by senior residents, who perform the entire consultation. Patients are staffed with the attending present that day who provides feedback, oversight, and supervision and helps the residents refine their clinical skills of indicating a patient for surgery, pre-operative planning, and post-surgical follow-up visits.

Dedicated clinic front office staff streamline the process by assisting with surgical quotes, preoperative clearance, and operating room scheduling. The resident aesthetic clinic does not engage in any formal advertising, but rather relies on word-of-mouth and direct referrals for patient recruitment. Patients are billed on a fee-for-service basis and insurance is not accepted. The revenue generated is used to offset operational costs of the clinic and operating rooms, as well as contribute to the salary of staff supporting the clinic. The remaining operational costs of the clinic are subsidized by the department.

We conducted a retrospective review of all surgical and injectable procedures completed by the NYU Resident Aesthetic Surgery Clinic in 2021. Patients were excluded from the study if outcome information was missing. Participating residents were surveyed before and after their rotation in the clinic to assess their confidence performing aesthetic procedures which was rated on a scale of 1 to 5.

Procedures were defined similar to how procedures are recorded for resident case logs for the Accreditation Council for Graduate Medical Education (ACGME).^1^ Bilateral procedures (*e.g.*, bilateral breast augmentation) were counted as two separate procedures (left and right). Blepharoplasty was not only separated by laterality, but also by upper and lower lids. In contrast, an operation was defined as a single trip to the operating room or office visit in which the patient received at least one procedure. Complications were categorized as either minor, moderate, or major. Minor complications were treated with non-operative management. Moderate complications required a small intervention such as seroma aspiration, while major complications necessitated return to the operating room. Complication and revision rates were calculated by summing the number of complications and revisions, respectively, and dividing by the total number of procedures.

Data analyzed using IBM SPSS software and the ggplot2 package in R. A chi-squared test assessed for significant deviation in frequencies of procedures across yearly quarters. Mann-Whitney tests evaluated significance between pre- and post-rotation confidence ratings among residents. A p-value of less than 0.05 was considered statistically significant. Complication and revision rates were calculated with respect to the total number of procedures. The complication rate, revision rate, and surgeon fees were compared to data from other institutions available in the literature. Surgeon fees were also compared to national averages according to the 2020 ASPS Plastic Surgery Statistics Report and 2021 Aesthetic Plastic Surgery National Databank Statistics.

**Results/Complications:**

In 2021, 144/379 consultations led to an operation (38.0% conversion rate), resulting in 420 distinct surgical procedures. The majority (53.3%) of procedures involved the head and neck. Complication and revision rates were 5.5% and 1.0%, respectively, with surgeon fees consistently below national average. Residents reported being significantly more confident performing face lifts, rhinoplasties, and aesthetic surgery in general following their clinic rotation. Analyzing our injectable experience, 223/233 consultations led to an injection (95.7% conversion rate) for 142 patients. Neurotoxin was most commonly injected into the upper face, while fillers were most commonly injected into the midface. Only 2 complications from fillers (0.9% complication rate) and one touch-up (0.4% touch-up rate) occurred. Procedural fees were substantially less than national averages and comparable to other academic settings.

**Conclusion:**

These data represent the largest annual reported study of plastic surgery resident aesthetic surgical and injectable procedures and outcomes, demonstrating the high volume and productivity of the NYU Resident Aesthetic Surgery Clinic. These results further support resident aesthetic clinics as a robust training modality.

